# Diastolic dysfunction and mortality in early severe sepsis and septic shock: a prospective, observational echocardiography study

**DOI:** 10.1186/2036-7902-4-8

**Published:** 2012-05-04

**Authors:** Samuel M Brown, Joel E Pittman, Eliotte L Hirshberg, Jason P Jones, Michael J Lanspa, Kathryn G Kuttler, Sheldon E Litwin, Colin K Grissom

**Affiliations:** 1 Division of Pulmonary and Critical Care Medicine, University of Utah School of Medicine, Salt Lake City, UT, 84132, USA; 2 Division of Pulmonary and Critical Care Medicine, Intermountain Medical Center, Murray, UT, 84107, USA; 3 Critical Care Echocardiography Service, Intermountain Medical Center, Murray, UT, 84107, USA; 4 Division of Critical Care, Department of Pediatrics, University of Utah School of Medicine, Salt Lake City, UT, 84108, USA; 5 Research and Evaluation, Southern California Permanente Medical Group, Pasadena, CA, 91101, USA; 6 Homer Warner Center for Informatics Research, Intermountain Healthcare, Salt Lake City, UT, 84107, USA; 7 Division of Cardiology, Georgia Health Sciences Health System, Augusta, GA, 30912, USA

**Keywords:** Sepsis, echocardiography, Diastolic dysfunction, Shock

## Abstract

**Background:**

Patients with severe sepsis or septic shock often exhibit significant cardiovascular dysfunction. We sought to determine whether severity of diastolic dysfunction assessed by transthoracic echocardiography (TTE) predicts 28-day mortality.

**Methods:**

In this prospective, observational study conducted in two intensive care units at a tertiary care hospital, 78 patients (age 53.2 ± 17.1 years; 51% females; mean APACHE II score 23.3 ± 7.4) with severe sepsis or septic shock underwent TTE within 6 h of ICU admission, after 18 to 32 h, and after resolution of shock. Left ventricular (LV) diastolic dysfunction was defined according to modified American Society of Echocardiography 2009 guidelines using E, A, and e’ velocities; E/A and E/e’; and E deceleration time. Systolic dysfunction was defined as an ejection fraction < 45%.

**Results:**

Twenty-seven patients (36.5%) had diastolic dysfunction on initial echocardiogram, while 47 patients (61.8%) had diastolic dysfunction on at least one echocardiogram. Total mortality was 16.5%. The highest mortality (37.5%) was observed among patients with grade I diastolic dysfunction, an effect that persisted after controlling for age and APACHE II score. At time of initial TTE, central venous pressure (CVP) (11+/- 5 mmHg) did not differ among grades I-III, although patients with grade I received less intravenous fluid.

**Conclusions:**

LV diastolic dysfunction is common in septic patients. Grade I diastolic dysfunction, but not grades II and III, was associated with increased mortality. This finding may reflect inadequate fluid resuscitation in early sepsis despite an elevated CVP, suggesting a possible role for TTE in sepsis resuscitation.

## Background

Cardiovascular dysfunction is a central component of the multiple organ dysfunction syndrome, an often fatal sequela of severe sepsis and septic shock. Although most research on cardiovascular dysfunction in septic shock has focused on left ventricular (LV) systolic dysfunction [1-5], LV diastolic dysfunction also occurs [6,7]. In sepsis, cardiac dysfunction reflects both intrinsic dysfunction and the adequacy of loading conditions, including both preload and afterload. Intrinsic LV diastolic dysfunction may make patients more sensitive to volume expansion interventions, often termed fluid resuscitation and often guided by central venous pressure (CVP). This sensitivity to resuscitation may be particularly prominent among patients with more severe LV diastolic dysfunction, which is generally associated with elevated left-sided filling pressures.

**Figure 1:**
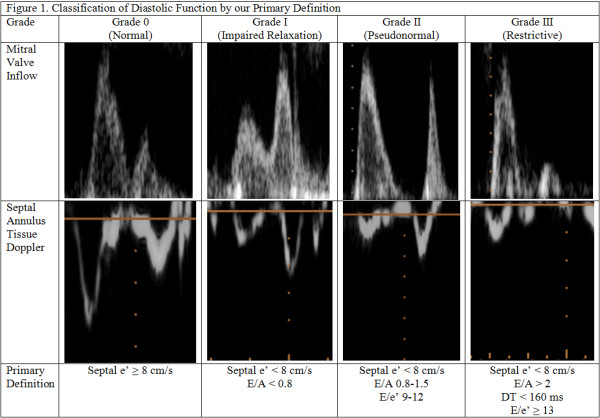
Classification of diastolic function by our primary definition.

LV diastolic function is traditionally classified into four grades (Figure 1) primarily using spectral Doppler of mitral inflow and tissue Doppler of the mitral annulus. In normal diastolic physiology, blood flow into the left ventricle occurs primarily during the early phase of diastole, resulting in the peak mitral inflow velocity during early diastole (E) being greater than the peak mitral inflow velocity of the atrial phase (A). The waveform of grade I diastolic dysfunction demonstrates E velocity less than A velocity as a reflection of impaired LV relaxation. Grade II waveforms are pseudonormalized (E greater than A) due to the opposing effects of impaired tissue relaxation and elevated left atrial pressure. Grade III waveforms display an E velocity much greater than A because atrial contraction is insufficient, in the face of an elevated left atrial pressure/volume, to propel blood into the noncompliant left ventricle. Tissue Doppler of the mitral annulus is considered largely preload independent and is a surrogate for the left ventricular relaxation rate during diastole [8]. As opposed to mitral inflow E velocity, the peak velocity of the mitral annulus during early diastole (e’) demonstrates a monotonic response to worsening intrinsic diastolic function, with e’ becoming slower with increasing severity of diastolic dysfunction. Moreover, the E/e’ ratio has been shown to correlate with LV filling pressures in many patient populations [9-11], including those with septic shock [12].

LV diastolic dysfunction is common and well characterized in community-dwelling adults with cardiovascular disease and is associated with significant morbidity and mortality in patients with either normal or reduced systolic function [13-16]. Much less is known about LV diastolic dysfunction in severe sepsis and septic shock. Previous studies on LV diastolic dysfunction in patients with severe sepsis and septic shock have employed various definitions and have not yielded a stable estimate of incidence [6,7,17-19]. Some reports suggest that the presence of LV diastolic dysfunction may be associated with poor outcome [20,21], while others suggest no effect on outcomes [17,18]. The natural evolution of diastolic dysfunction and its clinical implications during the course of sepsis and septic shock are also not well characterized.

We prospectively evaluated LV diastolic dysfunction among patients presenting to the ICU with severe sepsis or septic shock, with special emphasis on the different grades of diastolic dysfunction. We hypothesized that septic patients with more severe LV diastolic dysfunction would have worse outcomes.

## Methods

### Study design

This prospective, observational study was conducted between September 2008 and April 2010 at the Intermountain Medical Center, an academic, tertiary care hospital in Murray, UT, USA. Patients admitted to the 24-bed Shock Trauma Intensive Care Unit or the 12-bed Respiratory ICU were eligible for the study. The Intermountain Medical Center Institutional Review Board approved this study. All patients or their legally authorized representatives provided written, informed consent.

### Patients

Study investigators screened patients admitted to study ICUs with severe sepsis or septic shock defined by the American College of Chest Physicians/Society of Critical Care Medicine consensus criteria [22]. Patients met criteria for inclusion if (1) they were at least 14 years of age, (2) they had a suspected infection, (3) they had two or more systemic inflammatory response syndrome criteria, and (4) either (a) had severe sepsis (end-organ dysfunction) or (b) had septic shock (a systolic blood pressure less than 90 mmHg despite an intravenous fluid challenge of at least 20 ml/kg with evidence of organ dysfunction or hyperlactatemia. Exclusion criteria were primary diagnosis of acute coronary syndrome or major cardiac dysrhythmia, presence of pericardial tamponade, presence of mitral stenosis, known diagnosis of severe pulmonary hypertension, lack of sinus rhythm during initial echocardiogram, contraindication to central venous catheterization, or lack of commitment to intensive therapy.

Patients were treated according to the Surviving Sepsis Guidelines [23]. Specifically, in those patients requiring a central venous catheter, treatment followed an early goal-directed therapy protocol, targeting a mean arterial pressure of ≥ 65 mm Hg, CVP ≥ 8 mmHg, and central venous oxygen saturation ≥ 70% [24].

### Transthoracic echocardiography

Serial transthoracic echocardiograms (TTEs) were performed using either a Philips SONOS 5500, iE-33, or CX-50 (Philips Medical Systems, Bothell, WA, USA). We performed the first TTE within the first 6 h of admission to the ICU, the second at 18–32 h after admission, and the third at least 24 h after cessation of vasoactive medications or resolution of severe sepsis in patients who did not require vasoactive medications. Where a TTE was performed for clinical reasons, we analyzed the clinical TTE. In all other cases, investigators performed research TTEs, which were not employed in the clinical care of patients. All TTEs were interpreted by the second author, and a consensus interpretation was provided by one of two level-II echocardiographers (SMB, CKG) who are testamurs of the National Board of Echocardiography.

Diastolic function was assessed by measuring E and A peak velocities using spectral Doppler of mitral inflow and e’ and a’ velocities using tissue Doppler of the septal mitral annulus in the apical four-chamber view. Each data point represents the average of measurements from three consecutive cardiac cycles. In patients with sinus tachycardia, E and e’ were determined by previously described methods [25-27]. Mitral deceleration time (DT) and left atrial (LA) area at end systole were also measured. We defined diastolic dysfunction according to the American Society of Echocardiography (ASE) 2009 guidelines [28], classifying subjects into grade 0 (normal) if e’ ≥ 8 cm/s; grade I (impaired relaxation) if e’ < 8 cm/s and E/A < 0.8; grade II (pseudonormal) if e’ < 8 cm/s, E/A 0.8-1.5, and E/e’ 9–12; and grade III (restrictive) if e’ < 8 cm/s, E/A > 2, DT < 160 ms, and E/e’ ≥ 13 (Figure 1). The ASE guidelines were modified in that LA size was not used to define diastolic function because LA enlargement is most likely a slowly developing adaptation to chronically elevated LV filling pressures and not a reflection of acute diastolic dysfunction. In a sensitivity analysis, we evaluated the definition of diastolic dysfunction proposed by Sturgess et al. [19]. LV systolic dysfunction was defined as an LV ejection fraction (EF) < 45% [5].

### Clinical data

CVP was measured at the time of each TTE if the patient had a central venous catheter in place. Vital signs, mechanical ventilation parameters, and doses of vasoactive medications were recorded. Current intravenous fluid administration rate as well as total volume infused and total urine output in the period leading up to the TTE were obtained. All-cause inpatient mortality, ICU-free days, and ventilator-free days [29] at 28 days for all enrolled patients were determined. APACHE II scores at the time of ICU admission and for the day on which each TTE was performed were calculated [30]. Patient demographics and body mass index were also recorded at the time of study enrollment.

### Primary analysis

We assessed the incidence of LV diastolic dysfunction on first or subsequent TTE as well as the relationship between LV diastolic dysfunction on the initial TTE and 28-day outcomes. Our primary outcome was 28-day mortality, with secondary outcomes of ventilator-free days and ICU-free days.

### Secondary analyses

We evaluated the evolution of diastolic function through time and the relationship with inpatient mortality, ventilator-free days, and ICU-free days. We also evaluated the concordance of various proposed definitions of diastolic dysfunction. We also evaluated the incidences of LV systolic and isolated diastolic dysfunctions.

### Statistical methods

Central tendencies were compared using Fisher's exact test, Student's t test, Wilcoxon rank-sum, and Kruskal-Wallis test where appropriate. Where ties made Wilcoxon rank-sum problematic, we employed a permutation technique equivalent to one-way analysis of variance (R package coin). Regression models were built to predict mortality, ventilator-free days, and ICU-free days. All analyses were performed using the R Statistical Package (2.11.0) [31].

## Results

### Clinical and demographic findings

**Figure 2:**
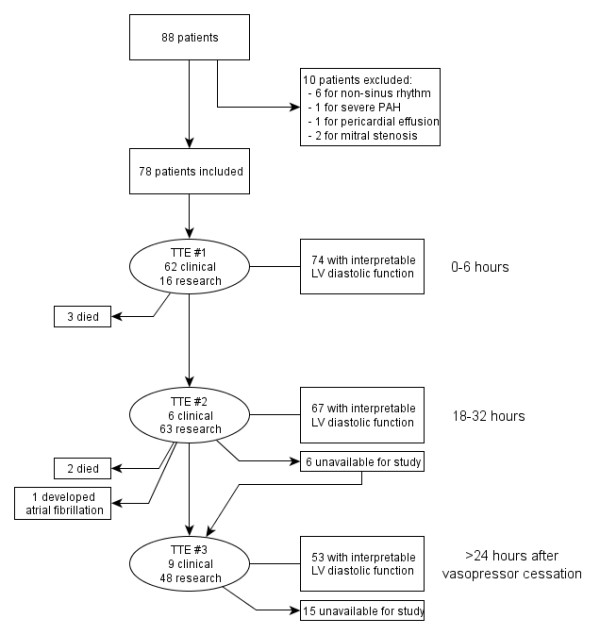
Flow of patients within the study.

We enrolled 78 patients in the study, as outlined in Figure 2. Demographic and clinical information regarding patients are displayed in Table 1. Overall mortality in the cohort was 16.5%. Forty-four percent of subjects required mechanical ventilation, and 64% required vasopressor therapy.

**Table 1:** Demographics and clinical findings

**Characteristic**	**Value**
Patients (*n*)	78
Age (years ± SD)	53.2 ± 17.1
Female (%)	51.3
APACHE II (score ± SD)	23.3 ± 7.4
Ever received vasopressor infusion (%)	64.1
Ever received mechanical ventilation (%)	43.6
MAP at admission (mmHg, median, IQR)	71 (65–79)
Highest PEEP (cm H_2_0)	11.4
Mortality (%)	16.5
Source of infection (%):	
Thorax (pneumonia)	35.9
Urinary tract	14.1
Abdomen	11.5
Skin/soft tissue	10.3
Blood stream	6.4
Central nervous system	1.3
Endocarditis	1.3
Multiple sources	6.4
Unidentified	12.8

APACHE II, Acute Physiology, Age, Chronic Health Evaluation score; BMI, body mass index; IQR, interquartile range; MAP, mean arterial pressure; PEEP, positive end-expiratory pressure; SD, standard deviation.

### Echocardiographic measurements

Two hundred four TTEs were performed on the 78 patients. The initial echocardiogram was performed in all 78 patients. The second echocardiogram was performed in 69 of 75 patients still alive and in the hospital on the second hospital day, while the third echocardiogram was performed in 57 of 73 patients who remained alive and in the hospital. Due to the inherent limitations of TTE, not all patients had evaluable diastolic dysfunction. Nonetheless, complete diastolic function was evaluable in 74 of 78 patients (94.8%) on the first echocardiogram, in 67 of 69 patients (97.1%) on the second, and in 53 of 57 patients (93.0%) on the third.

### LV diastolic dysfunction and clinical outcomes

Patients with grade I diastolic dysfunction on their initial echocardiogram had higher mortality (Table 2) versus all other patients (OR 1.28, p = 0.06), an effect that became significant after controlling for age greater than 50 years (OR 1.34, p = 0.03) in multivariate logistic regression. Among patients with diastolic dysfunction (grades I, II, or III; N = 27), grade I diastolic dysfunction was associated with higher mortality (OR 1.33, p = 0.03), even after controlling for age greater than 50 years, receipt of vasoactive medications, and APACHE II score upon admission.

**Table 2:** Incidence and clinical characteristics of diastolic dysfunction by ASE guidelines on initial echocardiogram

**Characteristic**	**Grade 0 (normal)**	**Grade I (impaired relaxation)**	**Grade II (pseudonormal)**	**Grade III (restrictive)**	**p value (any difference among groups)**	**p value (grade I vs grades II and III)**
Patients (N, %)	47 (64%)	8 (11%)	17 (23%)	2 (3%)		
Median age (years)	53	64	54	47	0.03	0.01
Female (%)	44.7%	62.5%	58.8%	50.0%	0.72	1.0
APACHE II	21.0	25.0	25.0	23.5	0.16	0.79
Percent on vasopressors	40.1%	25.0%	35.3%	50.0%	0.89	0.68
BMI (kg/m^2^)	28.3	26.4	33.8	32.9	0.31	0.07
e’ (cm/s)	9.97	5.82	7.16	3.99	<0.01	0.04
E/e’	9.1	12.0	12.5	26.7	0.01	0.67
DT (ms)	151	169	187	123	0.28	0.67
E/A	1.16	0.72	0.93	3.30	<0.01	<0.01
IVC diameter (cm)	1.90	1.91	1.94	2.18	0.85	0.89
IVC collapsibility (%)	32.7%	27.0%	23.6%	11.1%	0.18	0.55
Cardiac output (L/min)	6.4	4.0	6.5	4.2	0.10	0.45
VFD (days)	20.5	17.2	23.6	26.5	0.76	0.34
ICU-free days	17.9	15.8	18.9	25.5	0.98	0.96
Mortality (%)	12.8%	37.5%	5.9%	0%	0.19	0.06
IVF administered prior to TTE #1 (L)	3.5	2.6	5.5	5.4	0.07	0.05
IVF administered in 24 h after TTE #1 (L)	4.0	2.7	3.2	5.5	0.3	0.6
CVP (mm Hg)	11	11	12	11	0.89	0.84

APACHE II; Acute Physiology, Age, Chronic Health Evaluation score; BMI, body mass index; e’, early diastolic peak velocity of septal mitral annulus using tissue Doppler imaging; E, early diastolic mitral inflow velocity using spectral Doppler; A, atrial peak mitral inflow velocity using spectral Doppler; DT, E deceleration time; IVC, inferior vena cava; VFD, ventilator-free days; IVF, intravenous fluids; CVP, central venous pressure; TTE, transthoracic echocardiogram.

Patients with grade I diastolic dysfunction had similar CVPs (p = 0.89) compared to patients with grade II or higher diastolic dysfunction (Table 2). Patients with grade I diastolic dysfunction received significantly less volume expansion prior to the initial echocardiogram than patients with grade II or higher diastolic dysfunction (p = 0.05). Amount of volume expansion after initial echocardiogram did not differ among the different grades of diastolic dysfunction.

### Incidence of diastolic dysfunction

Forty-seven of 76 patients (61.8%) in whom diastolic function could be determined had LV diastolic dysfunction on at least one echocardiogram. Twenty-seven patients (36.4%) had LV diastolic dysfunction on their initial echocardiogram, while 33 patients (55.9%) had LV diastolic dysfunction on the final echocardiogram (Table 3). Different definitions of diastolic dysfunction yielded different estimates of incidence (Table 3). Notably, the ASE 2009 requirement to include measures of LA size would have excluded most of our study patients, as LA enlargement is an indicator of chronicity of diastolic dysfunction [32]. The incidence of isolated diastolic dysfunction (excluding patients with systolic dysfunction) upon presentation was 23.6% (Table 4).

**Table 3:** Incidence of diastolic dysfunction by serial TTE and by definition employed

**Definition**	**TTE #1 (0–6 h)**			**TTE #2 (18–32 h)**			**TTE #3 (post-shock)**		
**Definition of diastolic dysfunction**	**Total abnormal**	**Total measured**	**Percentage abnormal (%)**	**Total abnormal**	**Total measured**	**Percentage abnormal (%)**	**Total abnormal**	**Total measured**	**Percentage abnormal (%)**
Primary definition	27	74	36.4	30	68	44.1	33	59	55.9
Full ASE definition	1	70	1.4	0	55	0	1	50	2.0
e’ < 9.6 cm/s or E/e’ >15 (Fischer et al. [14])	44	74	59.4	44	61	72.1	40	57	70.2

TTE, transthoracic echocardiogram; ASE, American Society of Echocardiography; e’, early diastolic peak velocity of septal mitral annulus using tissue Doppler imaging; E, early diastolic mitral inflow velocity using spectral Doppler.

**Table 4:** Clinical and echocardiographic findings based on presence of systolic and/or diastolic dysfunction

**Characteristic**	**Normal**	**Systolic dysfunction (LVEF < 45 %)**	**Isolated diastolic dysfunction (grades I, II, and III)**	**p value**
Patients (N, %)	36 (50 %)	19 (26.4 %)	17 (23.6 %)	
Age (years, mean)	51.6	50.9	58.6	0.21
Female (%)	50 %	47.4 %	64.7 %	0.53
APACHE II (mean)	22.0	25.9	25.3	0.03
BMI (kg/m^2^, median)	28.6	26.9	28.9	0.45
e’ (cm/sec, median)	9.97	8.06	7.05	<0.01
E/e’	9.7	12.3	11.6	0.31
DT (ms, median)	156	129	180	0.003
E/A (median)	1.17	0.94	0.87	0.03
VFD (days, mean)	19.4	10.5	26.7	0.72
ICU-free days (median)	23.9	23.6	23.6	0.93
28-day mortality (mean)	13.9 %	10.5 %	17.6 %	0.83

Clinical and echocardiographic findings among patients with normal, impaired systolic,and isolated impaired diastolic function, on initial echocardiogram. APACHE II, Acute Physiology, Age, Chronic Health Evaluation score; BMI, body mass index; e’, early peak velocity of the septal mitral annulus using tissue Doppler imaging; E, early peak mitral inflow velocity using spectral Doppler; A, atrial peak mitral inflow velocity using spectral Doppler; DT, mitral deceleration time; VFD, ventilator-free days.

### Evolution of diastolic dysfunction

Of the 27 patients with LV diastolic dysfunction on initial echocardiogram, 9 (33%) had normal diastolic function on their final echocardiogram. Of the 47 patients with normal diastolic function on their initial echocardiogram, 11 (23.4%) displayed LV diastolic dysfunction on the final echocardiogram. Patients whose initial LV diastolic dysfunction improved by the third echocardiogram were younger than patients whose LV diastolic dysfunction persisted (38 years vs 63.5 years, p < 0.01). Patients who developed LV diastolic dysfunction between the first and last echocardiogram were not significantly older (54 years vs 52 years) than those whose LV diastolic function remained normal. Neither development nor resolution of LV diastolic dysfunction over the course of severe sepsis or septic shock was associated with a difference in mortality, ventilator-free days, or ICU-free days.

## Discussion

This study is, to our knowledge, the largest prospective evaluation of LV diastolic dysfunction by TTE in patients admitted to the ICU for severe sepsis or septic shock. LV diastolic dysfunction is relatively common during severe sepsis and septic shock with 34.6% and 61.8% of the cohort showing evidence of LV diastolic dysfunction on admission or at some point during their clinical course of sepsis, respectively. Notably, grade I diastolic dysfunction (generally felt to indicate a relatively low left atrial pressure) was associated with worse outcome when compared with grade II or III diastolic dysfunction. This novel finding was not consistent with our original hypothesis.

The current study is in conflict with a single previous study that suggested worse outcome with more severe LV diastolic dysfunction [21]. The reason for this discrepancy may be related to patient population, treatment algorithms, timing of the TTE, or definition of diastolic dysfunction employed. Our study specifically suggests that among patients presenting to the ICU with impaired diastolic function, those with low filling pressures as assessed by TTE have worse outcome than patients with LV diastolic dysfunction and higher filling pressures. The epidemiology and implications of diastolic dysfunction during critical illness appear to be quite different from that among community dwellers with stable disease or patients with acute cardiovascular disease (decompensated congestive heart failure or myocardial infarction). A similar effect has been observed in LV systolic function with a lower left ventricular ejection fraction associated with better survival in sepsis [1,4], although there was no difference in mortality in our cohort of patients with normal versus low LV systolic function.

Rivers and colleagues' pivotal study of early aggressive optimization of oxygen delivery in patients with severe sepsis or septic shock suggested that initial resuscitation was crucial to outcome [24]. That pivotal study, however, relied upon CVP to assess adequacy of volume expansion, a measurement that has been criticized on grounds that CVP poorly predicts an increase in cardiac output after volume expansion [33-35]. In our study, the mean CVP among those patients with grade I diastolic dysfunction was the same as that among patients with grade II or III diastolic dysfunction. Our study suggests that there may exist a group of patients with grade I diastolic dysfunction on TTE who would benefit from further volume expansion despite an elevated CVP. While small numbers in the subgroup of patients with grade I diastolic dysfunction limit generalizability, the higher mortality and lesser volume expansion prior to ICU admission suggest that further volume resuscitation in this group may improve survival. This study may provide equipoise for an interventional study of echocardiographic versus CVP-driven volume expansion among patients with early severe sepsis or septic shock.

Our study also highlights the need to establish a consistent, reproducible definition of LV diastolic dysfunction among critically ill patients, especially those with severe sepsis or septic shock. Prior studies report a varied incidence of LV diastolic dysfunction in patients with severe sepsis or septic shock, which may reflect a difference in patient populations and different definitions of diastolic dysfunction. In a retrospective review of 94 general ICU patients, Sturgess et al. reported an incidence of LV diastolic dysfunction equal to 67% [19]. Applying Sturgess and colleagues' definition to our cohort, we found a relatively consistent incidence of LV diastolic dysfunction on the initial echocardiogram of 61.7%. In a small study of 35 patients with septic shock requiring mechanical ventilation, Etchecopar-Chevreuil et al. found that LV diastolic dysfunction occurred in 20% of patients [18]. Bouhemad et al. reported a 20% incidence of isolated LV diastolic dysfunction in 54 post-operative patients with septic shock using transesophageal echocardiography [17]. Left atrial size is often used as a criterion for diagnosing LV diastolic dysfunction [28]. However, left atrial enlargement is most likely a slowly developing adaptation to chronically elevated LV filling pressures [32]. In the case of sepsis, diastolic abnormalities are frequently acute, and LA enlargement is not expected to occur rapidly. Therefore, LA size is not likely to be a reliable marker of diastolic filling abnormalities in this specific condition. Because of this, we chose to rely on the Doppler assessment of LV function and filling in this study.

The natural history of LV diastolic dysfunction in our cohort from the time of ICU admission to resolution of sepsis was variable, as some patients developed diastolic dysfunction over the course of their sepsis and others resolved their diastolic dysfunction. This finding is incongruent with other studies reporting that diastolic dysfunction in sepsis is transient and reversible [17,18]. Due to the continually changing physiologic profile of patients with severe sepsis and septic shock, the clinical importance of this finding is unclear. Future studies with classification of patients by age and grade of diastolic dysfunction should help clarify the evolution and clinical implication of LV diastolic dysfunction in severe sepsis and septic shock.

The limitations of this study include its observational nature. We used both research and clinical TTEs for our analyses. Clinicians were blinded to the research TTEs, but the clinical TTEs may have influenced clinical management. However, fluid management after the initial echocardiogram, which was similar regardless of degree of diastolic dysfunction, does not suggest that knowledge of the TTE changed management. Additionally, we chose to define diastolic dysfunction by the ASE 2009 guidelines, which were not developed for patients with sepsis. Furthermore, we specifically measured tissue Doppler of the septal annulus which may overestimate the severity of diastolic dysfunction [36]. While other measures of diastolic function are available, such as the indexed left atrial volume and/or the ratio of the diastolic reversal in pulmonary inflow to mitral A inflow duration [37,38], pulmonary inflow is difficult to obtain reliably in critically ill patients, and in our cohort, left atrial dilatation was extremely rare. The relatively small number of patients in patient subgroups underscores the importance of much larger sample sizes for studies in critical care echocardiography than have been reported to date. In addition, the study investigators are level-II echocardiographers. Whether the same observations would obtain in healthcare settings with less-trained critical care echocardiographers is not known, although the markers of diastolic dysfunction investigated in this study are generally easy to learn.

Two strengths of this study are its prospective nature and its size: it is one of the largest studies to date of critical care echocardiography. We were able to obtain acceptable images and quantify LV diastolic function in 95% of these critically ill patients, many of whom are generally considered to have poor windows for TTE, suggesting that TTE is useful in critically ill patients. TTE has major advantages over transesophageal echocardiography in terms of risk to the patient and ease in performing the exam, particularly for serial studies.

## Conclusions

LV diastolic dysfunction occurs frequently during severe sepsis and septic shock. Grade I diastolic dysfunction is associated with increased mortality when compared to either patients with normal filling patterns or those with grade II or III diastolic dysfunction. The association of worsening outcome with the less severe form of diastolic dysfunction could suggest the deleterious effect of inadequate volume expansion at the time of ICU admission. Our findings suggest that TTE may identify patients who require further fluid resuscitation during severe sepsis and septic shock despite a CVP that indicates adequate resuscitation according to current guidelines. Future clinical studies randomly comparing the use of CVP versus TTE for assessing the adequacy of fluid resuscitation are needed to confirm these findings. Further work to standardize the definitions of diastolic dysfunction in critically ill patients is also needed.

## Abbreviations

A: atrial diastolic mitral inflow velocity; ASE: American Society of Echocardiography; CVP: central venous pressure; DT: deceleration time; E: early diastolic mitral inflow velocity; e’, early diastolic septal mitral annulus velocity; LA: left atrium/left atrial; LV: left ventricle/left ventricular; TTE: transthoracic echocardiogram.

## Competing interests

The authors declare that they have no competing interests.

## Authors' contributions

SMB participated in the study design, data acquisition, data analysis, and manuscript writing, and approved the publication of this study. JEP participated in study design, data acquisition, data analysis, and manuscript writing, and approved the publication of this study. ELH participated in study design, data acquisition, and manuscript revision for important intellectual content, and approved the publication of this study. JPJ participated in study design, data analysis and manuscript revision for important intellectual content, and approved the publication of this study. ML participated in data acquisition and manuscript revision for important intellectual content, and approved the publication of this study. KK participated in data acquisition and manuscript revision for important intellectual content, and approved the publication of this study. SL participated in study design and manuscript revision for important intellectual content, and approved the publication of this study. CG participated in study design, data acquisition, and manuscript writing, and approved the publication of this study. All authors’ read and approved the final manuscript.
